# Modeling and Optimization of α‑Amylase Immobilization on Chitosan-Based Supports: A Comparison of Response Surface Methodology and Machine Learning Approaches

**DOI:** 10.1021/acsomega.6c01339

**Published:** 2026-04-07

**Authors:** Başak Birdal, Beyza Bahçıvan, Kübra Akbulut, Fatih Tarlak, Barış Binay

**Affiliations:** 1 Department of Bioengineering, 52962 Gebze Technical University, Kocaeli 41400, Türkiye; 2 BAUZYME Biotechnology Co., Gebze Technical University Technopark, Kocaeli 41400, Türkiye

## Abstract

α-Amylases are among the most important enzymes used in various industries; however, their large-scale application is restricted by limited stability, low reusability, and low activity loss during operation. In this study, immobilization of α-amylase from *Priestia megaterium* (*Pm*Amy) onto glutaraldehyde-activated chitosan beads (*Pm*Amy@GA–CS) was optimized using response surface methodology (RSM) and machine learning (ML) approaches, including support vector regression (SVR), Gaussian process regression (GPR), and random forest regression (RFR). The effects of the CS concentration, GA concentration, and enzyme binding time were investigated. RSM analysis identified optimal immobilization conditions at 2.6% CS, 0.5% GA, and 6 h of binding time, resulting in approximately 90% immobilization efficiency and high enzymatic activity. ML models captured similar immobilization trends, with GPR exhibiting the highest predictive accuracy (*R* ^2^ ≈ 0.98) and superior generalization performance compared to SVR, RFR, and RSM. Immobilization produced nearly uniform porous beads and induced a shift in the pH optimum toward slightly alkaline conditions (from pH 7.0 to 8.0), while the temperature optimum remained unchanged at 40 °C. Furthermore, the immobilized enzyme exhibited significantly improved stability, with a 2.1-fold increase in thermal stability and enhanced storage stability. Moreover, *Pm*Amy@GA–CS retained approximately 50% of its initial activity after ten consecutive reuses, demonstrating strong operational stability. Overall, this study highlights the complementary strengths of the RSM and ML in designing robust immobilized enzymes and provides a solid foundation for the development of reusable biocatalysts.

## Graphical abstract

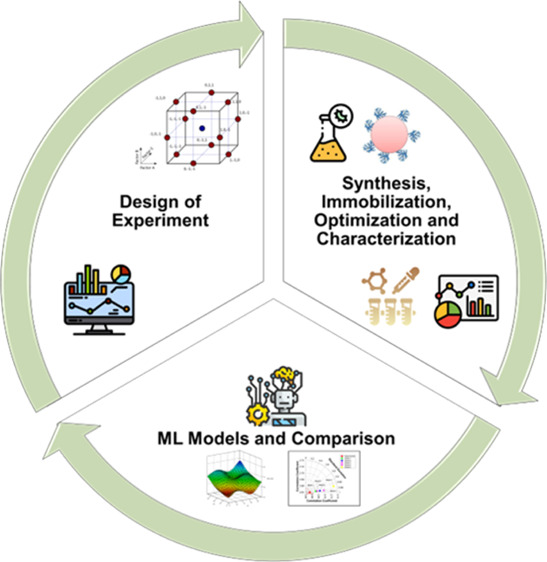

## Highlights

- *Pm*Amy was successfully immobilized on GA-CS beads and optimized using RSM and ML for the first time.
- Optimum conditions (2.6% CS, 0.5% GA, 6h binding time) achieved ∼90% immobilization efficiency.
- GPR outperformed RSM and other ML models, providing the best predictive accuracy (*R* ^2^ ≈ 0.98).
- Immobilization improved thermal stability, increasing the enzyme’s half-life by a factor of 2.1.
- The biocatalyst presented remarkable reusability, retaining 50% initial activity after 10 reuses.

## 1 Introduction

Biological catalysts of enzymes are crucial for different biochemical and biotechnological processes due to catalyzing specific reactions under defined conditions without the need for extreme pH or temperatures.^1^ Enzymes usually catalyze a one-step process depending on their substrate specificity and reaction efficiency as a result of limited byproduct formation.^2^ They have gained enhanced prominence in industrial applications, including chemical manufacturing, food processing, pharmaceuticals, and fine chemicals, as sustainable and environmentally friendly alternatives to chemical catalysts.^3^ However, free enzymes usually suffer from low solubility and stability, denaturation at high temperatures or in the presence of organic solvents, reduced catalytic activity, and substrate or product inhibition.^4^ These limitations not only restrict enzyme performance but also render their industrial usage economically unviable due to continuous enzyme replacement and enhanced operational costs.^5^ Therefore, enzyme immobilization, which is a technique to sequestrate or localize enzymes onto a support, has emerged as an efficient procedure to address these limitations.^6^

The chemical immobilization method, known as covalent bonding, is formed through reactions between the functional groups of the support and the enzyme.^7,8^ The main side chains of the enzyme that generate stable covalent complexes with support are cysteine (thiol group), aspartic acid, glutamic acid (carboxylic group), and lysine (amino group), while functional groups of supports are generally the imidazole, thiol, indole, hydroxyl, amino, and sulfhydryl groups.^7,9^ Activation of the support surface that acts as a bridge between the support and enzyme through covalent interactions is essential. Some of the multifunctional activating reagents are GA, tannic acid (TA), divinyl sulfone (DVS), carbodiimide, glycidol, epichlorohydrin, and formaldehyde.^10–13^ GA is a cheap, five-carbon aliphatic molecule widely used as a cross-linker due to its interaction with different regions of enzymes.^14,15^ It allows the generation of a molecular spacer between the support and enzyme molecule, thus minimizing the structural distortion while improving mechanical features and biosorption capacity.^16,17^ Hence, GA-activated supports are versatile and can generate covalent attachments, ion exchange, and hydrophobic adsorption.^12,18^ Between various supports, CS is widely preferred together with GA in immobilization studies, but their combination ratio, functionality duration, and usage for enzyme immobilization should be precisely optimized.

CS is one of the most abundant polysaccharides that is manufactured from one of the richest renewable natural resources of chitin. CS is structurally a copolymer of *N*-acetyl-d-glucosamine and d-glucosamine^19^ and has usage in several applications like medicine, agriculture, wastewater, and biotechnology due to its attractive properties such as nontoxicity, biodegradability, biocompatibility, ease of structural modification, thermal and chemical stability, and low cost.^20–22^ Due to its reactive functional groups of hydroxyl (−OH) and amino (−NH_2_), its properties can be chemically modified to give it new properties according to the intended application.^23^ Thus, they have emerged as a preferred support for enzyme immobilization that facilitates covalent coupling or adsorption. Its versatility in fabrication, enabling beads, films, fibers, and other geometries, further enhances its applicability across diverse biocatalytic processes. Consequently, CS-based immobilization systems have garnered extensive research interest and have been widely adopted in industrial applications, but optimization studies using tools are limited.^24–26^

Immobilization efficiency is critically governed by parameters, such as contact time, pH, temperature, enzyme loading, and support concentration. Conventional optimization techniques, such as one-factor-at-a-time (OFAT), have weaknesses such as being costly and time-consuming. They also fail to reveal complex interactions between variables that determine immobilization yield.^27^ Hence, design and engineering of immobilization processes have evolved to mathematical modeling and optimization techniques such as factorial designs, Plackett-Burman, and RSM. Between these techniques, RSM provides a robust framework for modeling and analyzing multifactorial systems to optimize performance responses. By fitting empirical models, RSM enables both the prediction of process outcomes and the identification of critical factor interactions.^28^ Among RSM techniques, the Box–Behnken Design (BBD) is especially beneficial due to its facilitation of effective estimation of quadratic model parameters, providing sequential experimentation, integrating blocking factors, and allowing strict lack-of-fit testing. Based on three-level incomplete factorial arrangements, a nearly rotatable design is constructed in BBD by strategically excluding experimental runs in which all factors simultaneously assume their maximum or minimum levels. Therefore, it reduces the risk of performing trials under potentially extreme or detrimental conditions.^29,30^ Despite these strengths, RSM-based models are intrinsically constrained by their dependence on predefined polynomial functions, which may be inadequate for accurately representing highly nonlinear and complex relationships among process variables. Enzyme immobilization processes, particularly those involving covalent cross-linkers such as GA, are influenced by heterogeneous enzyme orientations, diffusion limitations, time-dependent bond maturation, and microenvironmental effects, all of which introduce nonlinearities and interactions that deviate substantially from classical polynomial assumptions.

In this context, ML has emerged as a powerful alternative and complementary modeling approach, gaining increasing attention in biotechnological sciences. ML algorithms have recently been employed to effectively learn complex nonlinear interactions among process variables without requiring explicit mathematical assumptions, thereby offering enhanced predictive and modeling capabilities.^31^ These algorithms can provide robust modeling of complex data sets that pose challenges for traditional statistical approaches. Common ML models utilized in biotechnological studies include SVR, linear regression (LR), gradient boosting decision trees (GBDT), random forests (RF), GPR, and artificial neural networks (ANN).^32–34^ Selecting suitable methods is crucial and is typically achieved through comparative evaluation of model performance metrics such as root-mean-square error (RMSE), coefficient of determination (*R* ^2^), and cross-validation scores on the experimental data set.^35^

In this study, RSM and ML were employed as the core modeling and optimization frameworks to immobilize a bacterial α-amylase onto glutaraldehyde-activated chitosan (GA-CS) beads. Critical process parameters, including GA-CS concentration and enzyme–support contact time, were systematically optimized using both approaches to comparatively assess the predictive accuracy and modeling performance of statistical versus data-driven computational methods. Following model-based optimization, the resulting *Pm*Amy@GA-CS biocatalysts were subjected to comprehensive physicochemical and catalytic characterization with Fourier Transform Infrared Spectroscopy (FTIR) and Scanning Electron Microscopy (SEM) analyses, together with pH/temperature optima, thermal and storage stability, and reusability analyses.

## 2 Materials and Methods

### 2.1 Microorganism, Chemicals, and Equipments

*Escherichia coli* ( *E. coli* ) BL21 (DE3) cells, which were transformed with *Pm*Amy, including pET-28b­(+) vector and chitosan, were obtained from Gebze Enzyme Recognition Center. (GERC, Gebze, Türkiye). GA (25%) was kindly requested from the Department of Chemistry of Gebze Technical University (Gebze, Türkiye). 3,5 – Dinitrosalicylic acid (DNS) 98% was obtained from Acros Organics. BCA Protein Assay Kit was obtained from Thermo Fisher Scientific Inc. (Waltham, Massachusetts, USA). Five mL Ni^2+^-NTA His-Trap and PD-10 columns were purchased from Cytiva, Amersham, UK. Autoinduction Studier autoinduction medium was from Formedium (Formedium, Swaffham, England). Other chemicals used during the study were obtained from Sigma-Aldrich (StLouis, MO, USA).

### 2.2 Optimization Using BBD

The experiments were designed and analyzed using BBD of RSM for the optimization of factors that affect the immobilization process. Preliminary experiments were conducted to determine the initial values of the factors (data not shown). Effects of immobilization factors were assessed using a 3-level 3-factor experimental design composed of 15 trials. The three factors and their levels are as follows: CS concentration (2.0, 2.5, and 3.0%), GA concentration (0.5, 1.0, and 1.5%), and enzyme binding time (6, 16, and 24 h). Both for the experimental design and evaluation of the results statistics, Minitab Statistical Software was used.^36^ The mathematical correlation between the variables in the responses can be specified as in eq 1.

$$Y_{i}=\beta_{0}+\Sigma^{\beta i}X_{i}+\Sigma^{\beta ii}{X^{2}}_{i}+\Sigma^{\beta ij}X_{i}X_{j}$$ 1

where *Y_i_* represents the predicted responses while *X_i_X_j_* are input variables affecting the response of *Y*; β_0_ is the offset; β_*i*_ is the *i* ^th^ linear coefficient; β*_ii_* is the *i* ^th^ quadratic coefficient; and β*_ij_* is the *ij* ^th^ interaction coefficient. In the study, 15 sets of experiments were carried out to obtain the coefficients. Model validation was conducted under the determined optimal conditions.

### 2.3 ML Models

The predictive power of an ML model is determined by the extent to which bias and variance are balanced for a particular data set. SVR, a nonparametric, kernel-based extension of support vector machines, is employed to project the data into a higher-dimensional space, allowing both linear and nonlinear relationships to be captured.^37^ In this study, the radial basis function (RBF) kernel was applied, as it is known to perform effectively in high-dimensional contexts, although sensitivity to noisy data can be observed.

RFR builds predictive models by combining the outputs of many decision trees, each trained on a different bootstrap sample of the data. This ensemble approach is well-suited to capture complex, nonlinear relationships while remaining adaptable to previously unseen data. By relying on a forest of trees rather than a single tree, RFR reduces the tendency to overfit and allows more reliable interpretation of nuanced predictor–response dynamics.^38,39^

GPR is considered a fully Bayesian and nonparametric alternative.^40^ Within this structure, the target function is designed as a multivariate Gaussian distribution, allowing the derivation of both mean predictions and credible intervals. Applying a squared-exponential kernel, smooth and continuous trends are obtained, and robust predictions are produced even in sparsely sampled regions. Still, the inversion of a full covariance matrix is needed, which makes the method computationally expensive for large-scale data sets.

In this study, into the data set including 45 observations (15 experiments × 3 replicates), a resampling strategy was implemented. The original data were resampled at random, and uniform noise, which was described using the interquartile range to the standard deviation of the three replicates, was added to the target variable across the entire data set, resulting in 750 augmented samples. This procedure efficiently increased the sample size and avoided overfitting. Nevertheless, it is recognized that the introduction of label noise is analogous to generating synthetic values and may modify the underlying error structure, potentially inflating the perceived robustness and interval coverage. Following augmentation, the preprocessed data were divided into training and testing subsets as a ratio of 80:20. Data, models, and obtained plots can be obtained from the following GitHub link: PmAmy_Immobilization_RSM_ML.

### 2.4 *Pm*Amy Purification

Synthesis of *Pm*Amy was achieved as explained in our previous work.^41^ Briefly, cells were precultured in 5 mL of LB (Luria–Bertani) with a final kanamycin concentration of 50 μg μL^–1^, grown at 37 °C and 180 rpm for overnight. Enzyme synthesis was started by inoculating 1% of the preculture into 400 mL Studier autoinduction media and cultured at 25 °C and 180 rpm for 24h. After growth, cells were harvested using a centrifuge at 4000 rpm and 4 °C for 20 min.

For the purification of intracellular *Pm*Amy, the pellet was resuspended in a buffer including 20 mM sodium phosphate (pH 7.4), 500 mM NaCl, 30 mM imidazole, supplemented with 0.5 mg mL^–1^ lysozyme and 0.5 mM phenylmethylsulfonyl fluoride (PMSF), and incubated on ice for enzymatic cell lysis. Then, suspension was subjected to mechanical cell disruption of sonication at 50% power amplitude with 20 cycles of 15 s with 15 s intervals. The lysate was clarified by centrifugation at 11,000 rpm for 45 min at 4 °C and filtered through 0.45 μm filters to be used as an overexpressed protein source. The supernatant was loaded into the 5 mL pre-equilibrated Ni-NTA His-Trap affinity column, and bound *Pm*Amy was eluted with 100- and 200-mM imidazole, including phosphate buffers. Eluted fractions were combined and desalted by using a PD-10 column with 0.05 M sodium phosphate buffer (pH 7.0).

### 2.5 Immobilization of *Pm*Amy on GA-CS Beads

#### 2.5.1 Preparation of CS Beads

CS powder was dissolved in 2% (v/v) acetic acid under mild heating for 1 h. The resulting solution was then stored at 4 °C to facilitate the removal of the entrapped air bubbles. The bubble-free CS solution was added dropwise to 15 mL of 1 M NaOH solution using a 2 mL syringe to form beads. The beads were then gently stirred in the same solution at room temperature for 1 h to allow hardening. Subsequently, the CS beads were washed several times with ultrapure water to remove residual acidity.

#### 2.5.2 Activation of CS beads with GA

A commercial 25% GA solution was diluted to obtain final concentrations of 0.5, 1.0, and 1.5% (v/v) and mixed with CS beads at a ratio of 1:10 (w/v). The suspension was gently shaken at room temperature to facilitate surface activation. The GA-activated beads were then washed with 0.05 M sodium phosphate buffer (pH 7.0) and subsequently used for *Pm*Amy immobilization.

#### 2.5.3 Covalent Immobilization of *Pm*Amy on GA-CS Beads

The functionalized CS beads were incubated with 0.5 mg mL^–1^ *Pm*Amy solution in 0.05 M sodium phosphate buffer (pH 7.0) at a ratio of 1:10 (w/v) under gentle stirring at 4 °C for a predetermined time. Following immobilization, the beads were separated and washed several times to remove unbound *Pm*Amy. The residual enzyme concentration in the buffer solution was determined using the BCA assay.^42^ The amount of immobilized enzyme (IE, g g^–1^ of beads) was then calculated according to eq 2.^43^

$$\mathrm{IE}(g\;g^{-1}\;\mathrm{beads})=\frac{V_{x}(C_{i}-C_{f})}{m}$$ 2

where *V* is the volume of the *Pm*Amy solution at (mL), *C_i_* is the initial *Pm*Amy concentration (mg mL^–1^), *C_f_* is the final *Pm*Amy concentration at the end of immobilization (mg mL^–1^), and *m* is the mass of the support (g).

### 2.6 Enzyme Assays

#### 2.6.1 *Pm*Amy Activity Assay

The activity of soluble *Pm*Amy was determined by measuring the concentration of released reducing sugars using the DNS assay.^44^ For each reaction, 0.1 mL of free amylase was mixed with 0.1 mL of 1% (w/v) starch solution and incubated at 40 °C for 5 min. The reaction was terminated by adding 0.2 mL of DNS reagent, followed by incubation at 95 °C for 10 min. The absorbance was then measured at 540 nm.

#### 2.6.2 *Pm*Amy@GA-CS Activity Assay

*Pm*Amy@GA-CS beads suspended in 0.1 mL of 0.05 M sodium phosphate buffer (pH 7.0) were incubated with 0.1 mL of 1% (w/v) starch solution under constant stirring at 40 °C for 10 min. Following the reaction, the beads were removed from the mixture, and 0.2 mL of DNS reagent was added. The tubes were then incubated at 95 °C for 10 min, after which the absorbance was measured at 540 nm. One unit of amylase activity was defined as the amount of enzyme required to release 1 μmol of maltose per minute under the assay conditions. The specific activity (U g^–1^) of *Pm*Amy@GA-CS beads was determined by stating the relationship between the enzymatic activity and the amount of immobilized protein (eq 3).^45^

$$\mathrm{Specific}\;\mathrm{Activity}=\frac{\mathrm{EA}}{\mathrm{IE}}$$ 3

where EA is the enzymatic activity of the beads (U g^–1^ beads), and IE is the amount of immobilized enzyme (g enzyme g^–1^ of beads). Additionally, immobilization yield (IY) was calculated based on the initial enzymatic activity of *Pm*Amy and the residual enzyme activity of *Pm*Amy in the supernatant of the immobilization mixture, according to eq 4.^46^

$$\mathrm{IY}=\frac{({\mathrm{EA}}_{0}-{\mathrm{EA}}_{f})\times 100}{{\mathrm{EA}}_{0}}$$ 4

where EA_0_ is the initial enzyme activity (U g^–1^), and EA_*f*_ is the final enzyme activity in supernatant (U g^–1^) after the immobilization reaction.

### 2.7 Characterization

#### 2.7.1 FTIR and SEM analyses of CS, GA-CS, and *Pm*Amy@GA-CS Beads

FTIR analysis was carried out for the confirmation of amylase cross-linking on CS beads, as well as analyzing their chemical functionalization by recording spectra in the range of 500–4000 cm^–1^, each consisting of an average of 32 scans and a resolution of 4 cm^–1^. Surface morphology of CS beads, GA-activated CS beads, and *Pm*Amy@GA-CS beads was evaluated by SEM (Philips XL30 SFEG). SEM images were collected at an accelerating voltage of 15 kV and at varying magnifications, ranging from 65 to 20000x.

#### 2.7.2 Biochemical Characterization

The optimum working pH of free *Pm*Amy and *Pm*Amy@GA-CS beads was determined using a range of buffers: 0.05 M sodium citrate (5.0), 0.05 M sodium phosphate (pH 6.0–8.0), and 0.05 M glycine buffer (pH 9.0 and 10.0). The optimum reaction temperature of free and immobilized *Pm*Amy was investigated at temperatures ranging from 20 to 80 °C at 10 °C intervals.

Free *Pm*Amy and *Pm*Amy@GA-CS beads were stored in 0.05 M sodium phosphate buffer at 4 °C, and their storage stability was evaluated by measuring the retained enzymatic activity at 1 week intervals. Thermal stability tests were carried out at 40 °C by incubating both free and immobilized *Pm*Amy samples in 0.05 M sodium phosphate buffer (pH 7.0). The residual enzymatic activities were determined at specified time intervals. The inactivation constant (*k*_d_), half-life (*t*_1/2_), and stabilization factor (SF) were then calculated.^47^

#### 2.7.3 Reusability

The reusability of the *Pm*Amy@GA-CS beads was evaluated over 10 consecutive reaction cycles using the immobilized enzyme assay described above. After each cycle, the beads were rinsed with 0.05 M sodium phosphate buffer (pH 7.0) and transferred into a fresh reaction medium for the subsequent cycle. The residual activity after each cycle was expressed as a percentage of the initial activity measured in the first cycle, which was defined as 100%.

## 3 Results and Discussion

### 3.1 Immobilization of *Pm*Amy on the GA-CS Beads

An acidic condition is necessary for CS dissolution; therefore, acetic acid is used for this purpose. During dissolution, the amino groups of CS are protonated. After transferring into NaOH solution, protonated groups are neutralized, and then spherical and nearly uniform porous beads are formed (Figure 1a).^48^

**Figure 1:**
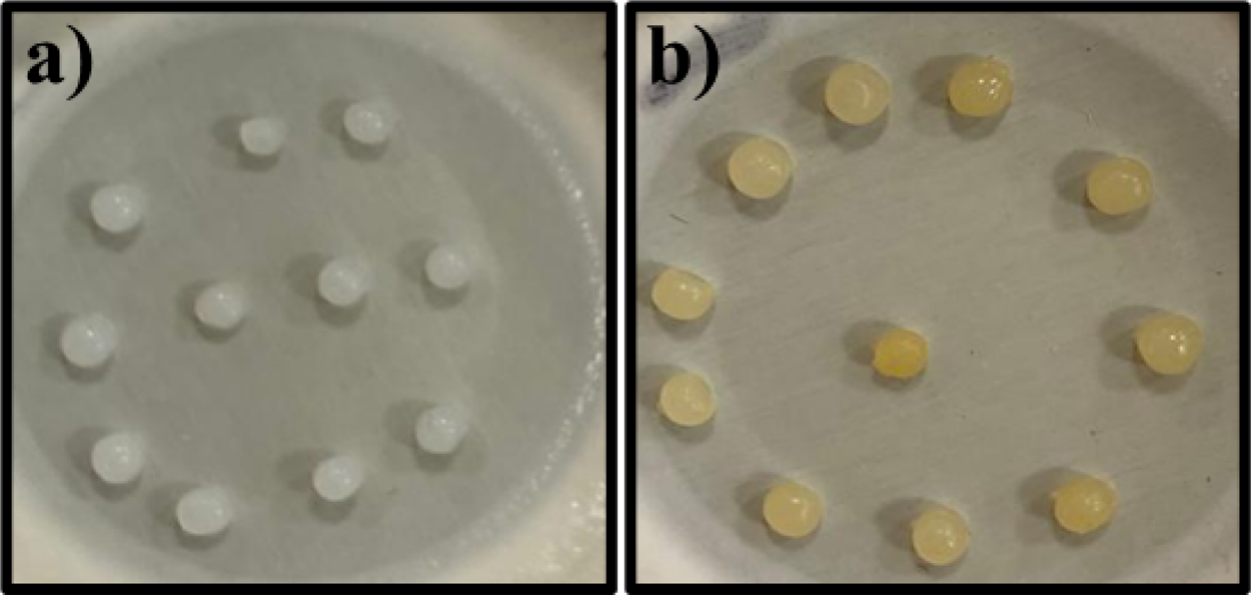
Form of beads. (a) Form of CS beads made in NaOH solution. (b) Form of CS beads activated with GA.

The cross-linking mechanism between CS and GA primarily involves the reaction of CS amino groups with GA aldehyde groups, generating imine (Schiff base, C=N) bonds that stabilize the polymeric matrix. However, not only does Schiff base formation occur, but GA can also undergo self-oligomerization through aldol condensation, causing extended polymeric chains within the cross-linking structure.^49^ Such secondary reactions have been suggested to increase the network rigidity and modify the microstructural characteristics of CS-based hydrogels and beads. An orange coloration observed during the cross-linking process corresponds to the presence of unsaturated bonds, including C=N, C=C, and C=O groups (Figure 1b).^49–52^

### 3.2 Optimization Using BBD

BBD was carried out to understand the relationship between process variables and determine the optimum immobilization conditions resulting in higher enzyme activity. Three scrutinized factors were CS concentration (*X*_1_), GA concentration (*X*_2_), and enzyme binding time (*X*_3_). Three levels of each factor were inspected along a range of values (+1 to −1). The detailed experimental parameters and enzyme activity results are given in Table 1.

**Table 1:** BBD for Optimization of *Pm*Amy on GA-CS Beads

				specific activity (Ug^‑1^)	
run	*X*_1_: CS concentration (%)	*X*_2_: GA concentration (%)	*X*_3_: binding time (h)	experimental	predicted
1	1 (3.0)	1 (1.5)	0 (16)	86	96
2	1 (3.0)	0 (1)	1 (24)	308	275
3	0 (2.5)	0 (1)	0 (16)	217	239
4	0 (2.5)	–1 (0.5)	1 (24)	480	482
5	–1 (2.0)	0 (1)	1 (24)	540	546
6	–1 (2.0)	0 (1)	–1 (6)	477	502
7	1 (3.0)	–1 (0.5)	0 (16)	214	235
8	0 (2.5)	1 (1.5)	1 (24)	473	488
9	0 (2.5)	–1 (0.5)	–1 (6)	690	672
10	0 (2.5)	0 (1)	0 (16)	222	239
11	–1 (2.0)	–1 (0.5)	0 (16)	366	352
12	0 (2.5)	1 (1.5)	–1 (6)	462	454
13	–1 (2.0)	1 (1.5)	0 (16)	329	304
14	0 (2.5)	0 (1)	0 (16)	284	239
15	1 (3.0)	0 (1)	–1 (6)	483	476

Experimental data was applied to multiple regression analysis, and a second-order polynomial equation was generated in terms of process variables as per eq 5. Statistical significance of the model was evaluated by ANOVA as presented in Table 2, and it demonstrated the significance of the acquired quadratic model with a *p*-value of 0.001.

$$\mathrm{Specific}\;\mathrm{Activity}\;(U/g)=238+812X_{1}-393X_{2}-72.6X_{3}-133{X_{1}}^{2}+164.0{X_{2}}^{2}+2.999.{X_{3}}^{2}-91.0X_{1}X_{2}-13.66X_{1}X_{3}+12.42X_{2}X_{3}$$ 5

**Table 2:** ANOVA for the RSM

**Source**	**DF** ^a^	**SS** ^b^	**MS** ^c^	***F*-value**	*p* **-value** ^d^	
Model	9	342,982	38,109	29.25	0.001	significant
Linear	3	78,302	26,101	20.04	0.003	
*X*_1_	1	43,788	43,788	33.61	0.002	
*X*_2_	1	22,423	22,423	17.21	0.009	
*X*_3_	1	12,090	12,090	9.28	0.029	
Square	3	223,829	74,610	57.27	0.000	
*X*_1_^2^	1	4082	4082	3.13	0.137	
*X*_2_^2^	1	6207	6207	4.76	0.081	
*X*_3_^2^	1	211,350	211,350	162.24	0.000	
2-way interaction	3	29,843	9948	7.64	0.026	
*X*_1_ *X*_2_	1	2070	2070	1.59	0.263	
*X*_1_ *X*_3_	1	15,206	15,206	11.67	0.019	
*X*_2_ *X*_3_	1	12,566	12,566	9.65	0.027	
Error	5	6513	1303			
Lack-of-fit	3	3727	1242	0.89	0.567	not significant
Pure error	2	2786	1393			
Total	14					

a Degree of freedom.

b Sum of squares.

c Mean square.

d Significant when *p* < 0.050.

With an insignificant lack-of-fit value of 0.567, the model fit the data well. The degree of variation around the mean that accounts for the data’s proximity to the fitted regression line is measured by the coefficient of determination (*R* ^2^). ANOVA yielded an acceptable value for *R* ^2^ of 0.98, indicating a very good association between the independent variables. The values of Adj-*R* ^2^ (0.95) and Pred-*R* ^2^ (0.81) were also in good agreement. According to the model, the most significant terms are the linear effect of all factors, the quadratic effects of binding time, and the linear relationship between CS concentration and binding time, together with GA concentration and binding time, due to offering the lowest *p*-values. Based on the results, optimum immobilization conditions of *Pm*Amy that correspond to a predicted specific activity of approximately 675 U g^–1^ were predicted to be obtained using 2.6% CS concentration, 0.5% GA concentration, and 6 h binding time.

Interactive effects of the parameters on *Pm*Amy immobilization were visualized by drawing response surface and contour plots (Figure 2).

**Figure 2:**
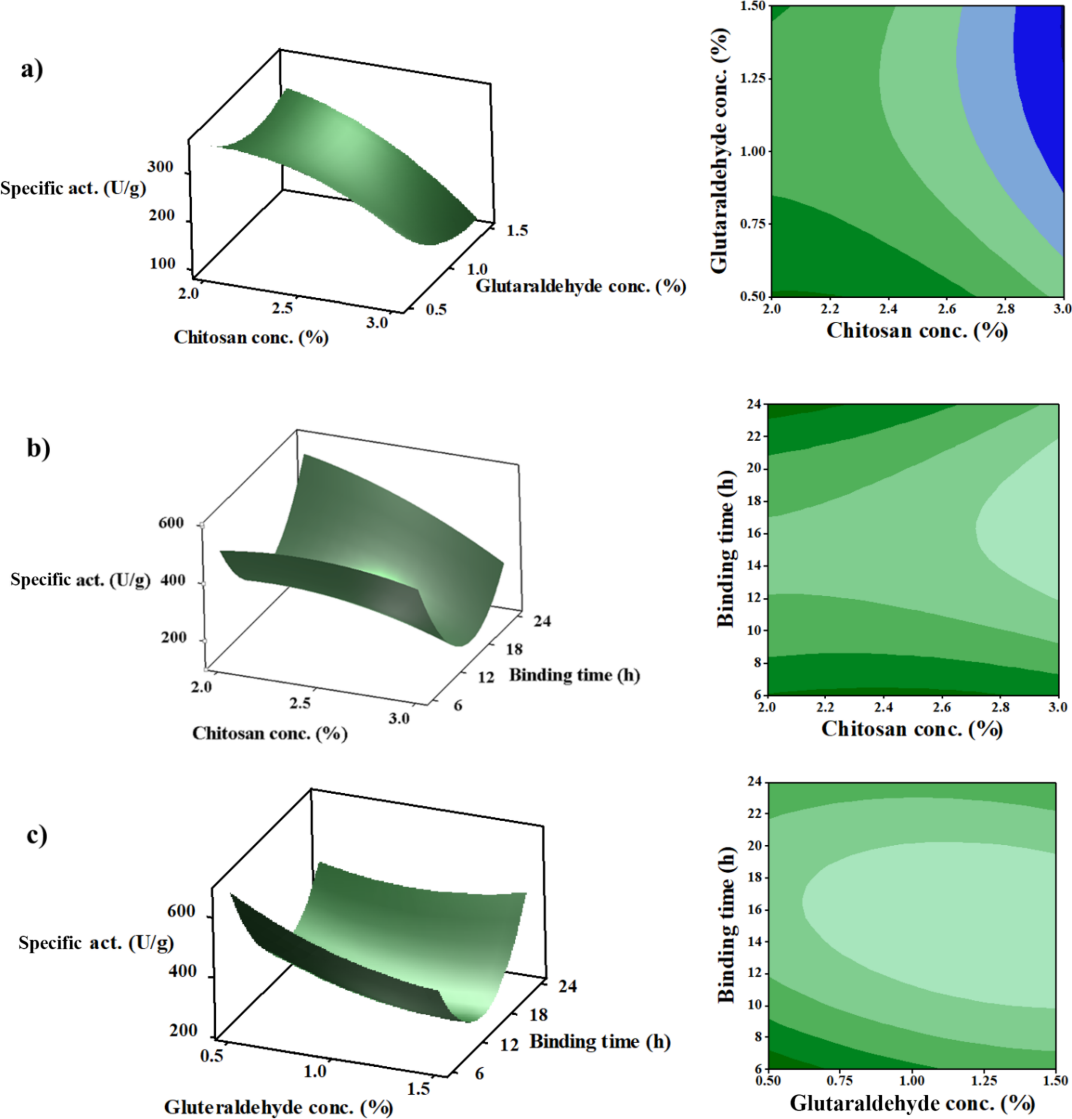
Response surface and contour plots showing the relationship between (a) CS and GA ratio, (b) CS ratio and binding time, and (c) GA ratio and binding time.

As illustrated in Figure 2a, enzyme activity increased with higher CS concentrations, reaching maximum levels between 2.0 and 2.6%. This enhancement can be attributed to improved bead integrity and porosity, which favor enzyme loading and mass transfer, but further enhancement causes a decrease in enzyme activity. In a previous study, carboxymethyl chitosan (CMCS) was used at concentrations ranging from 0.5 to 2.0% for the immobilization of alcalase while the alginate content was kept constant. The results showed that enzyme loading and specific activity increased with the CMCS concentration up to 1.5%, after which both parameters declined. This behavior was attributed to the fact that increasing the CMCS ratio initially enhances cross-linking with genipin and improves bead density; however, further increases in CMCS lead to excessive cross-linking, resulting in steric hindrance and restricted enzyme accessibility.^53^

However, when GA concentration increased, enzyme activity was negatively affected, mostly due to excessive cross-linking, which restricts conformational flexibility or blocks catalytic residues, as seen in the same trend in literature.^54,55^ For example, in the study conducted by Wang et al. (2014),^56^ a thermophilic esterase from *Archaeoglobus fulgidus* was first adsorbed onto a hydrophobic macroporous resin and subsequently treated with GA to minimize enzyme leaching and enhance operational stability. Increasing the GA concentration from 0.01 to 1.0% led to a pronounced decrease in the specific activity of the immobilized esterase, indicating that excessive cross-linking negatively affects catalytic performance. The authors reported that 0.01% GA was sufficient to effectively prevent enzyme leaching, while both 0.01 and 0.05% GA yielded immobilized preparations with a comparatively higher catalytic activity. Consequently, 0.05% GA was chosen for subsequent optimization experiments.^56^

For the response surface of CS concentration and binding time, a nonmonotonic trend in specific activity was observed. First, the activity was high at 6 h, dropped at intermediate times, and increased again by 24 h (Figure 2b). It could be explained as loosely adsorbed enzymes showed high catalytic activity, as binding time increased, suboptimal orientation and crowding occurred, causing diffusional and conformational constraints. Over longer times, maturation of imine linkages and the removal of weakly bound enzymes resulted in a more stable and productively oriented population, partially restoring activity.

Same with the first response surface, average GA levels (0.5–1.0%) together with lower binding times yielded the highest activity, while increasing GA concentration beyond this range consistently reduced activity, regardless of binding time (Figure 2c). This outcome highlights the delicate balance between ensuring enzyme-support attachment and preventing structural rigidity that compromises catalytic function.

Eventually, the RSM results denote that optimal immobilization is achieved at 2.6% CS concentration, 0.5% GA concentration, and 6 h binding time, leading to the highest α-amylase activity and about 90% immobilization yield. These findings align with previous reports demonstrating that fine-tuning immobilization parameters is crucial for maximizing enzyme performance.^57^

### 3.3 ML Models and Comparison with RSM

Three ML algorithms, SVR, GPR, and RFR, were employed to predict enzyme activity and determine the optimal immobilization conditions. Model performances were evaluated using both training and test data sets, and the corresponding statistical indicators are summarized in Table 3.

**Table 3:** ML Model Effect on Prediction of Enzyme Activity

model	process	***R*** ^2^	RMSE
SVR	train	0.977	22.972
SVR	test	0.983	20.681
GPR	train	0.981	20.758
GPR	test	0.985	19.569
RFR	train	0.981	20.781
RFR	test	0.985	19.512

All three models demonstrated strong predictive capability, with high coefficients of determination (*R* ^2^ > 0.97) and low prediction errors (RMSE ≈ 19–23), indicating their suitability for modeling the immobilization process. Among the evaluated models, GPR and RFR exhibited superior and consistent performance across both training and test data sets, achieving test *R* ^2^ values of approximately 0.985 with RMSE values close to 19.5. Although the SVR model also showed a high correlation, it consistently produced slightly higher RMSE values, suggesting comparatively reduced predictive precision. To further assess model behavior, Taylor diagrams were employed to simultaneously evaluate correlation, centered RMSE, and standard deviation between predicted and experimental values (Figure 3).

**Figure 3:**
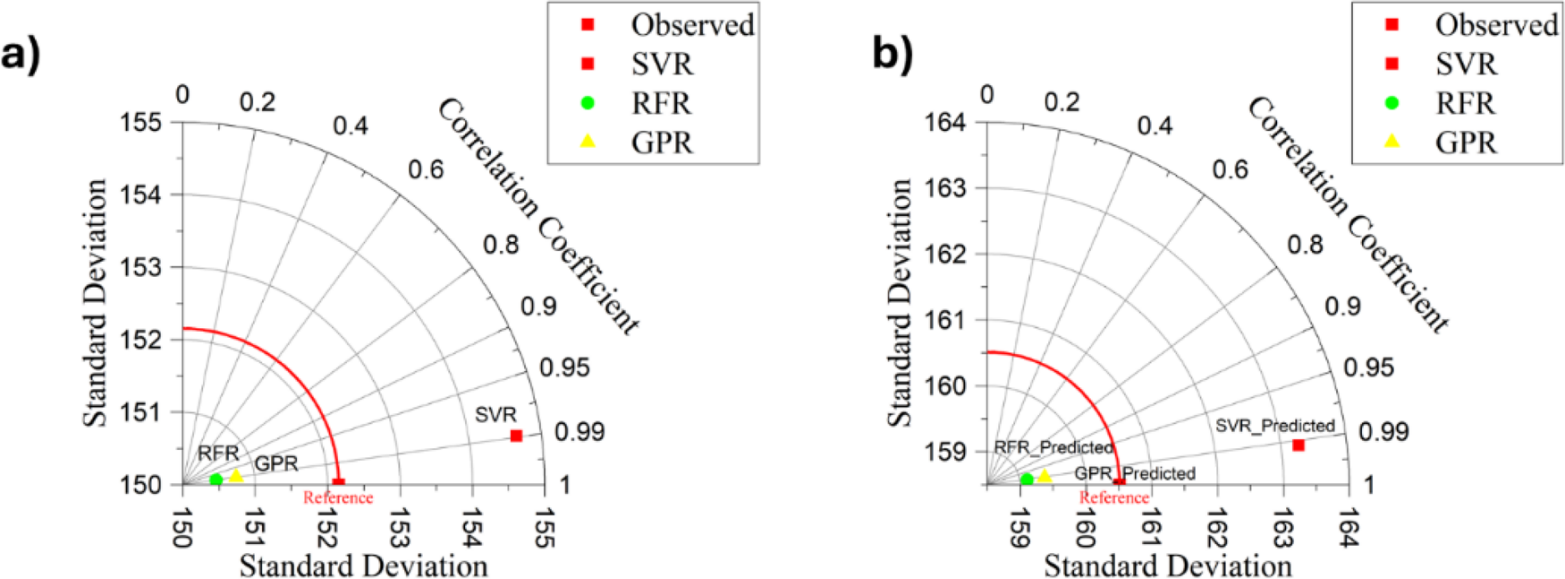
Taylor diagram for (a) training and (b) testing.

In both training and test data sets, GPR predictions were positioned closest to the reference point, indicating the lowest centered RMSE and the closest agreement with experimental variability. While RFR displayed predictive accuracy comparable to GPR. GPR was preferred due to its smoother response surface representation and its inherent ability to provide uncertainty-aware predictions, which is an important advantage when optimizing processes based on limited experimental data sets. Based on GPR modeling, the optimal immobilization conditions were identified as 2.51% CS, 0.5% GA, and 6 h binding time, corresponding to a predicted specific activity of approximately 692 U g^–1^.

A direct comparison between ML and RSM predictions revealed that both approaches captured consistent trends in α-amylase immobilization behavior. Importantly, the optimal immobilization conditions predicted by the RSM and ML were remarkably close, confirming the robustness and reliability of both optimization strategies. However, clear differences were observed in the predictive performance. Although the RSM model exhibited a high coefficient of determination (*R* ^2^ = 0.98), its predictive capability was comparatively limited, as reflected by a lower Pred-*R* ^2^ value (0.81). In contrast, ML models, particularly GPR, demonstrated superior generalization to unseen conditions with higher test *R* ^2^ values (∼0.985) and lower RMSE values (∼19.5).

The superior performance of GPR reflects the intrinsic physicochemical complexity of the immobilization process. Covalent attachment of *Pm*Amy onto GA-activated CS beads generates a heterogeneous population of enzyme molecules with varying orientations, degrees of multipoint attachment, and local diffusional environments. These factors introduce nonlinear responses and localized curvature in the process–response landscape that are difficult to adequately capture using second-order polynomial models. In contrast, GPR effectively learns such nonlinear dependencies directly from experimental data while preserving smoothness and providing uncertainty-aware predictions, making it particularly well-suited for enzyme immobilization systems characterized by limited yet information-rich data sets.

To the best of our knowledge, this study represents one of the few systematic comparisons between a classical statistical optimization method and multiple ML algorithms for enzyme immobilization optimization. Previous studies have similarly reported the superiority of ML-based approaches over RSM. For example, Fatiha et al.^58^ demonstrated that ANNs outperformed RSM in optimizing the immobilization of *Candida rugosa* lipase. Likewise, Hassan et al.^59^ showed that ANN-based optimization strategies provided enhanced robustness and predictive accuracy compared to RSM for the immobilization of pectinase and xylanase. With the increasing adoption of ML in enzyme engineering,^60–63^ the present findings further confirm that ML, GPR in particular, offers a powerful and reliable framework for modeling and optimizing complex enzyme immobilization systems.

### 3.4 Characterization

#### 3.4.1 FTIR and SEM

FTIR analysis was conducted to characterize the functional groups in CS beads, GA-CS beads, and *Pm*Amy@GA-CS beads to compare their characteristics (Figure 4).

**Figure 4:**
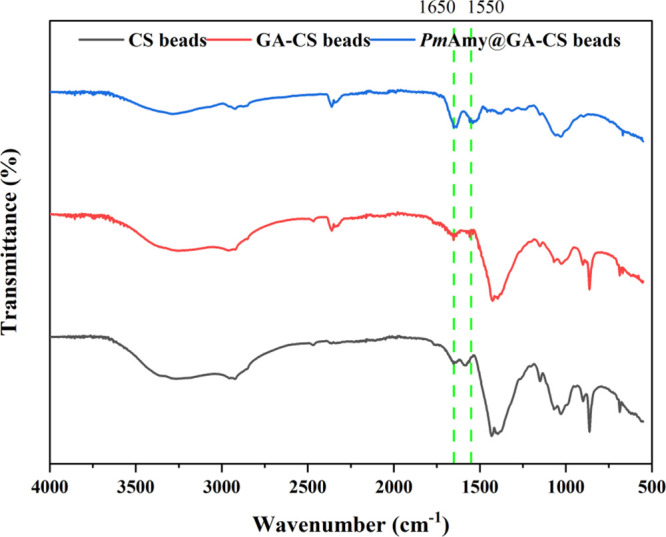
FTIR spectra of CS beads (black line), GA-CS beads (red line), and *Pm*Amy@GA-CS beads (blue line).

The native CS spectrum has well-defined bands between ∼1150 and 1030 cm^–1^ in the fingerprint region, associated with C–O–C and C–O stretching of the saccharide backbone, along with a weak signal near ∼870 cm^–1^ indicative of β-glycosidic linkages. Following GA activation (GA–CS), the band near ∼1643 cm^–1^ became more pronounced and slightly shifted relative to the original amide I region (∼1646 cm^–1^), reflecting the formation of Schiff base (C=N) linkages between GA and CS’s primary amine groups.^64,65^ After immobilization of *Pm*Amy, the spectrum of *Pm*Amy@GA–CS exhibited an intensified absorption near ∼1650 cm^–1^, attributed to overlapping contributions from both residual imine bonds and the protein’s amide I vibration. Additionally, a shoulder or slight increase in intensity was observed near ∼1540–1550 cm^–1^, corresponding to the amide II band, further confirming successful enzyme immobilization. Throughout the modification steps, the retention of key bands in the ∼1150–1030 cm^–1^ range demonstrated that the CS backbone remained structurally intact. Collectively, these spectral changes confirm effective GA cross-linking and covalent immobilization of *Pm*Amy on the CS support.^66,67^

SEM results of CS, GA-CS and *Pm*Amy@GA–CS beads were shown in Figure 5.

**Figure 5:**
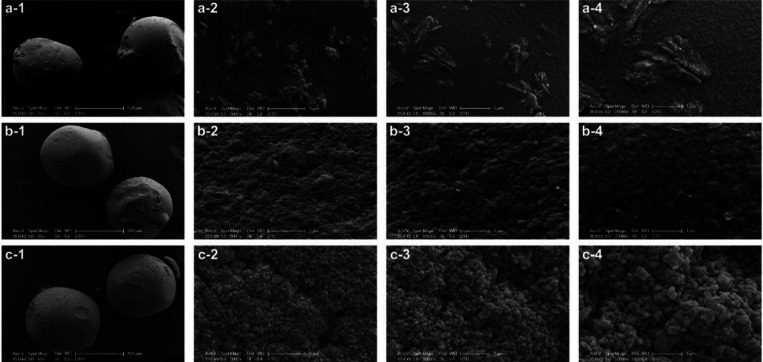
SEM images of (a) CS beads, (b) GA-CS beads, and (c) *Pm*Amy@GA–CS at varying magnifications, ranging from 65 to 20,000× (left to right).

SEM exhibited that native CS beads possessed smooth, uniform surfaces across all magnifications (Figure 5a-1–a-4). Following GA activation, the bead surfaces became noticeably rougher and more porous, consistent with cross-link formation (Figure 5b-1–b-4). After *Pm*Amy immobilization, GA–CS beads exhibited a markedly textured, aggregated morphology relative to CS and GA–CS. At higher magnifications (Figure 5c-2–c-4), discontinuous clusters and irregular, coarse granules formed a contiguous layer over the support, consistent with protein deposition and successful enzyme immobilization.

#### 3.4.2 Biochemical Characterization

Biochemical characterization of immobilized *Pm*Amy was analyzed against its free form. For optimum working pH determination, enzymes were incubated in various buffers having a pH range of 5 to 10, while temperature effects were observed between 20 and 80 °C. After determining the optimum temperature, enzymes were incubated for a period and remaining activity was measured to determine thermal stability. Results were represented in Figure 6.

**Figure 6:**
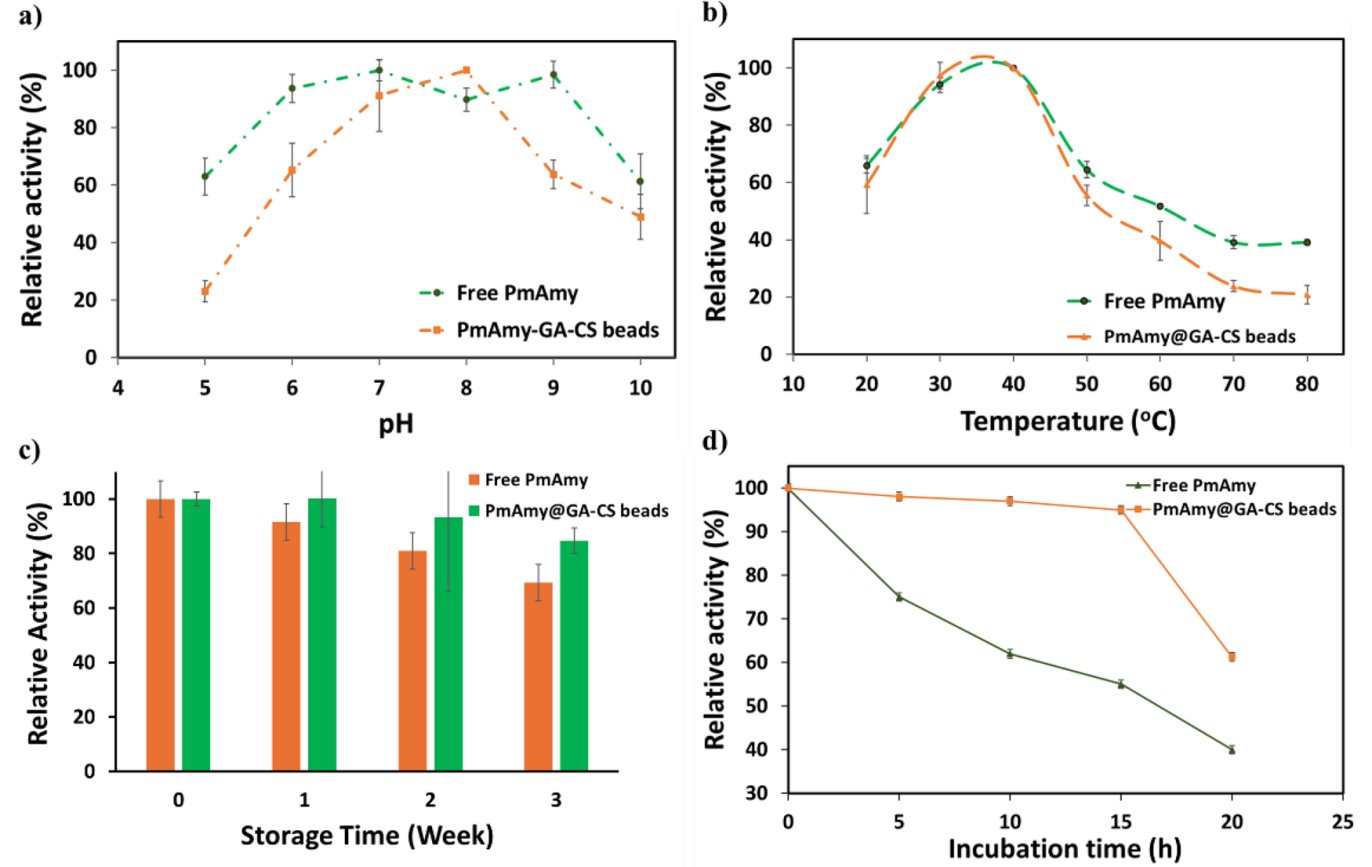
Biochemical characterization. (a) pH optima of free *Pm*Amy and *Pm*Amy@GA-CS, (b) temperature optima of free *Pm*Amy and *Pm*Amy@GA-CS, (c) thermal stability of free *Pm*Amy and *Pm*Amy@GA-CS at 40 °C for 20h, and (d) operational stability of free *Pm*Amy and *Pm*Amy@GA-CS during repeated incubation.

Based on biochemical characterization, the free *Pm*Amy exhibited maximal relative activity at pH 7, whereas the immobilized preparation *Pm*Amy@GA–CS showed an optimal pH of 8 (Figure 6a). Despite this shift, both forms retained substantial activity across a broad pH range under the tested conditions. The shift in pH optima through the alkaline level is attributable to a change in the microenvironment around the immobilized enzyme. While pH changes from acidic to neutral, protonated amino groups on CS lead to a positively charged layer that disturbs the ionization of acidic residues near the active site, thereby decreasing the catalytic efficiency. Consistent with this explanation, lipase immobilized on GA-activated CS beads displayed a similar shift in optimal pH from 6.5 to 8.^68^

On the other hand, immobilization did not change the temperature optima of *Pm*Amy as seen in Figure 6b. Along with this result, *Bacillus thuringiensis* HCB6 amylase, which was immobilized on CS beads, showed the same optimum working temperature as its free form (37 °C).^69^ On the other hand, even if t_opt_ remains unchanged, diffusional and microenvironmental constraints caused a reduction in the magnitude of activity at elevated temperatures.^70–72^ Storage stability tests were carried out for 3 weeks at 4 °C by incubating in sodium phosphate buffer. After this period, *Pm*Amy@GA–CS maintained about 85% of its initial activity, whereas the free enzyme retained ∼70%, demonstrating superior long-term stability conferred by immobilization (Figure 6c).

Following a thermal stability of 20 h at 40 °C, *Pm*Amy@GA–CS maintained higher than 50% of its initial activity, while the free *Pm*Amy retained about 40% (Figure 6d). These results verify that immobilization on GA-CS beads improved thermal stability at 40 °C. Additionally, the *k*_d_ values were obtained as 42.9 × 10^–3^ and 20.2 × 10^–3^ h^–1^, respectively (Table 4).

**Table 4:** Thermal Stability Parameters of Free and Immobilized *Pm*Amy at 40 °C

*Pm*Amy	*k*_d_ (h^–1^)	*t*_1/2_ (h)	SF
Free*Pm*Amy	42.9 × 10^–3^	16.2	1.0
*Pm*Amy@GA-CS beads	20.2 × 10^–3^	34.7	2.1

By using the *k*_d_ values, the corresponding *t*_1/2_ values were calculated as 16.2 and 34.7 h. These results denote that the thermal stability of *Pm*Amy@GA–CS was enhanced by a factor of 2.1, which highlights immobilization efficiency to enhance thermal stability. Covalent attachment of the enzyme on the CS matrix could restrict conformational mobility and, therefore, suppresses unfolding, resulting in enhanced thermal stability.^73,74^

#### 3.4.3 Reusability

Easily recoverable and reusable biocatalysts are attained with immobilization, which reduces costs associated with enzyme synthesis and purification. In the current study, the immobilized preparation of *Pm*Amy@GA–CS was reused across 10 successive reaction cycles under optimal conditions. *Pm*Amy@GA–CS preserved higher than 60% after 5 reuse, and it maintained approximately 50% of initial activity at the end of 10 reuse, indicating its high potential for industrial usage (Figure 7).

**Figure 7:**
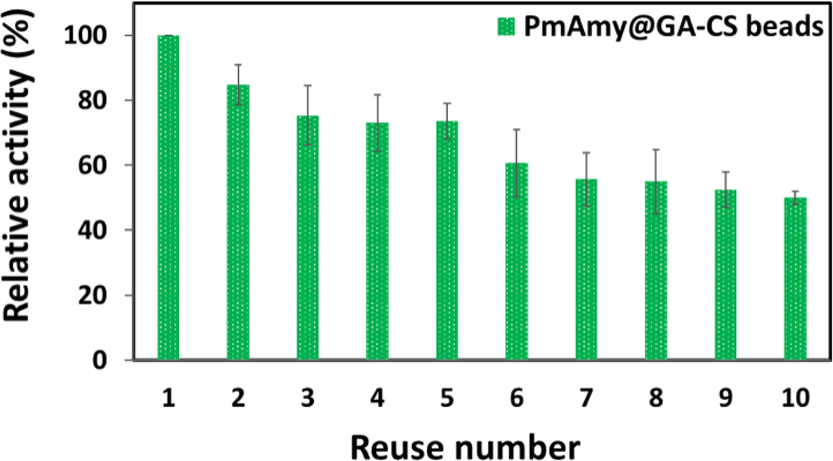
Reusability of *Pm*Amy@GA–CS for starch hydrolysis at 40 °C for 10 consecutive uses. The residual activity was calculated relative to the initial activity.

Similarly, a commercial amylase immobilized on unmodified CS beads preserved ∼50% of its initial activity after 10 consecutive reuse cycles, while immobilized on activated CS beads resulted in around 60% activity recovery.^75^ Another study also underscored the strong reusability potential of CS-based supports by testing an immobilized mannanase on GA-cross-linked CS beads for 15 consecutive reusability cycles and retained 54% of its initial activity.^67^

There are a few studies in which amylases were immobilized on CS beads, as listed in Table 5.

**Table 5:** Immobilization Studies of Amylases on CS Beads and Main Outcomes

amylase source	immobilization	findings	references
*P. megaterium*	covalent binding with GA	90% immobilization yield, higher thermal stability, and around 50% residual activity after 10 reuses	this study
*Canavalia gladiata*	covalent binding with GA	96% immobilization yield, high stability over a wide pH range, 76% residual activity after 10 reuse and 85% remained activity after 120 days of storage	^76^
Termamyl	multipoint covalent binding with GA together with lysine or asparagine	increase in immobilization yield, and stability in pH/temperature, operational, and stability after modifying with asparagine	^75^
*Bacillus thuringiensis*	covalent binding with tripolyphosphate (TPP)	%43 remaining activity after 5 reuse and enhanced substrate affinity	^69^
*Thermophilic Geobacillus*	covalent binding with GA	48 h stability at pH 9, 50% remaining activity at 95 °C for 48 h, and 50% remaining activity after 8 reuses	^77^
*Exiguobacterium sp.*	covalent binding with GA	yield of 56% with specific activity. Higher organic solvent tolerance	^78^
*Aspergillus carbonarius*	covalent binding with polyglutaraldehyde	improved thermal and storage stability, retained higher than 90% activity after 10 reuses	^79^

As can be seen in the Table 5, the general cross-linker agent that was utilized with CS is GA for covalent binding of amylases, resulting in more stable and at least 8 times reusable biocatalysts after immobilization. Covalent immobilization, achieved via GA cross-linking on CS beads, yields an amylase biocatalyst characterized by remarkably robust enzyme-support bonding, which not only prevents leaching to ensure high operational stability and reusability but also confers structural rigidity, which is essential for maintaining superior thermal tolerance.

## 4 Conclusions

In this study, α-amylase from *P. megaterium* was successfully immobilized onto GA–CS beads and systematically optimized by using RSM and ML approaches. RSM identified the optimal immobilization conditions as 2.6% CS, 0.5% GA, and 6 h binding time, yielding high enzymatic activity and approximately 90% immobilization efficiency, while ML, particularly GPR, provided superior predictive accuracy and identified very similar optimal conditions. Immobilization significantly enhanced enzyme performance by shifting the pH optimum toward alkaline values, preserving the temperature optimum, improving thermal and storage stability, and enabling effective reuse with ∼50% residual activity after 10 cycles. The close agreement between RSM and ML optimization results confirms the robustness of both approaches, while the higher predictive capability of GPR highlights the advantage of data-driven models in capturing nonlinear immobilization behavior. Overall, GA–CS beads represent an efficient and sustainable support for α-amylase immobilization, and the combined use of RSM and ML offers a powerful framework for designing stable, reusable biocatalysts with strong potential for industrial starch conversion and continuous bioprocess applications.

## Data Availability

The data supporting this study are available within the manuscript
